# Data mining-based recommendation system using social networks—an analytical study

**DOI:** 10.7717/peerj-cs.1202

**Published:** 2023-02-08

**Authors:** Sahar Ajmal, Muhammad Awais, Khaldoon S Khurshid, Muhammad Shoaib, Anas Abdelrahman

**Affiliations:** 1 Department of Computer Science, University of Engineering and Technology, Lahore, Punjab, Pakistan; 2 Department of Mechanical Engineering, Faculty of Engineering & Technology, Future University in Egypt, Cario, Egypt

**Keywords:** Social media, Data mining, Content-based filtering, Collaborative filtering, Recommendation system

## Abstract

In the current age, social media is commonly used and shares enormous data. However, a huge amount of data makes it difficult to deal with. It requires a lot of storage and processing time. The content produced by social media needs to be stored efficiently by using data mining methods for providing suitable recommendations. The goal of the study is to perform a systematic literature review (SLR) which finds, analyzes, and evaluates studies that relate to data mining-based recommendation systems using social networks (DRSN) from 2011 to 2021 and open up a path for scientific investigations to enhance the development of recommendation systems in a social network. The SLR follows Kitchenhem’s methodology for planning, guiding, and reporting the review. A systematic study selection procedure results in 42 studies that are analyzed in this article. The selected articles are examined on the base of four research questions. The research questions focus on publication venues, and chronological, and geographical distribution in DRSN. It also deals with approaches used to formulate DRSN, along with the dataset, size of the dataset, and evaluation metrics that validate the result of the selected study. Lastly, the limitations of the 42 studies are discussed. As a result, most articles published in 2018 acquired 21% of 42 articles, Whereas, China contributes 40% in this domain by comparing to other countries. Furthermore, 61% of articles are published in IEEE. Moreover, approximately 21% (nine out of 42 studies) use collaborative filtering for providing recommendations. Furthermore, the Twitter data set is common in that 19% of all other data sets are used, and precision and recall both cover 28% of selected articles for providing recommendations in social networks. The limitations show a need for a hybrid model that concatenates different algorithms and methods for providing recommendations. The study concludes that hybrid models may help to provide suitable recommendations on social media using data mining rules.

## Introduction

In the last two decades, social media has become popular and affordable for its users, making this world a global village. Social media is a digital media platform that users use for communicating and sharing content (Ilyas & Alharbi, 2022). The content can be personal activities, thoughts, pictures, news, ideas, debates, and government guidelines (Pang & Lee, 2008). Currently, Facebook, Twitter, Instagram, LinkedIn, YouTube, *etc.* are commonly used social media platforms (Kent, 2010; Howard & Parks, 2012) that produce data in linguistics (Kanwal, Qadir & Shaukat, 2021). The list of social networks is shown in Table 1. The social media platforms used in Table 1 provide facilities for online interaction, formation of social networks, and collaboration among users (Carr & Hayes, 2015). These platforms will be used in the future for collecting data and to perform mining in order to provide recommendations. Social media plays a significant role in sharing content online and formulating online social networks (Esmaeili & Vancheri, 2010). Therefore, social media is a group of applications that works over the Internet to exchange the content generated by users. The online produced content needs to be stored at some repositories to be utilized purposefully. Data mining is required to deal with this large, complex, and frequent data. Otherwise it is hard to extract meaningful information from social media (Hu & Liu, 2012; Gundecha & Liu, 2012). The data is represented in the form of nodes and edges during data mining; hence the nodes are considered users, and their interconnection is represented as links that formulate a graph-like structure (Chen et al., 2022). Data mining methods are used for information retrieval (Pang & Lee, 2008), statistical modeling (Koukouvinos, Mylona & Parpoula, 2013), and machine learning (Boiy & Moens, 2009; Alinejad-Rokny, Sadroddiny & Scaria, 2018). These methods firstly pre-process data (Baskar, Arockiam & Charles, 2013), then apply rules to analyze the data (Azzalini & Scarpa, 2012), and interpret data to obtain results (Smith et al., 2006). The result is represented in the form of graphs (Aggarwal, 2011). Data mining uses an unsupervised classification of data using semi-supervised classification and supervised classification (Simovici, 2015). Association rule mining is a type of data mining that helps to evaluate the data in a dataset that look for similarities or co-occurrences in data set (Alam et al., 2021) which make it easy to extract opinion from data (Shaukat et al., 2020a). After classification of data, the recommendation system (RS) (Nagarnaik & Thomas, 2015; Sharma & Gera, 2013) uses data mining rules for providing recommendations using state of the art recommendation methods; collaborative filtering (CF), content-based filtering (CBF) or hybrid model (Thorat, Goudar & Barve, 2015). It also uses data mining methods to provide recommendation on social network (Najafabadi, Mohamed & Mahrin, 2019; Faryal et al., 2015).

**Table 1:** Categories of social media platforms (Barbier & Liu, 2011).

**Category**	**Social sites**
Blogs	Blogger, LiveJournal, WordPress
Microblogs	Twitter, Google Buzz
Mining of opinion	Epinions, Yelp
Photo and video sharing	Flickr, YouTube, Pinterest, Netflix, Amazon Prime
Social bookmarking	Delicious StumbleUpon
Social networking sites	Facebook, Instagram, SnapchatLinkedIN, MySpace, Orkut
Social news	Digg, Slashdot
Wikis	Wikipedia, WikiHow, Scholarpedia, Event maps

The methods and techniques used for data mining based RS using social networks (DRSN) are large in number (Najafabadi, Mohamed & Mahrin, 2019; Faryal et al., 2015). It is difficult to identify which methods can provide recommendations by using social websites. Furthermore, different online platforms are available; therefore, every study uses data depending upon its requirement and uses evaluation methods accordingly. The overall research trend is also moving towards the data mining rule to provide recommendations, but it is still unambiguous (Vairavasundaram et al., 2015). A survey (Najafabadi, Mohamed & Mahrin, 2019; Anandhan et al., 2018); focuses on data mining methods in recommend systems. Another survey emphasizes data mining methods for social network analysis (Adedoyin-Olowe, Gaber & Stahl, 2013; Injadat, Salo & Nassif, 2016). Furthermore, there are existing SLRs (Alrashidi et al., 2021; Camacho & Alves-Souza, 2018) and surveys (Eirinaki et al., 2018; Javed et al., 2021) that focuses in the domain of RS. The existing survey (Najafabadi, Mohamed & Mahrin, 2019) deals with DRSN. However, it only highlights the studies that use CF methodology whereas (Anandhan et al., 2018) focuses on both data mining and RS methodologies, but it has explained them independently without linking their working. A comparison of existing studies in the domain of DRSN is exhibited in Table 2. It is extracted from Table 2 that there is scope for SLR in the domain of DRSN. So, there is a need for an SLR that focuses on all the domains, methodologies, and techniques used by different studies. This article contributes to propose a novel SLR that focuses on data mining rules on the social network for providing recommendations. The SLR deals with four research questions focusing on publication channels, type of research, chronological distribution, and geographical distribution. It also identifies proposed methods and most frequently algorithms that can provide better performance. Moreover, it focuses on the study of datasets, size of dataset and evaluation metrics used in the domain of DRNS. Lastly, limitations of existing work are discussed because the existing literature does not focus on all stated parameters on a single platform. Therefore, it is necessary to explore the details of DRSN.

**Table 2:** Comparison with existing literature to find research gaps.

References	Method	Data mining methods	Recommendation systems methods	Years analyzed for research	Libraries	Publication venues	Chronological distribution	Domain	Method	Dataset	Evaluation	Research gaps
Najafabadi, Mohamed & Mahrin (2019)	Survey	✓	✓	2010–2016	4	×	✓	×	×	✓	×	×
Anandhan et al. (2018)	Survey	✓	✓	2011–2015	2	×	×	✓	✓	✓	✓	✓
Adedoyin-Olowe, Gaber & Stahl (2013)	Survey	✓	×	Not mentioned	×	×	×	×	×	×	×	×
Injadat, Salo & Nassif (2016)	Survey	✓	×	2003–2015	7	×	×	✓	✓	×	×	✓
Javed et al. (2021)	Survey	×	✓	2000–2015	×	×	✓	×	✓	×	×	×
Alrashidi et al. (2021)	SLR	×	✓	2016–2020	6	×	×	×	✓	×	×	✓
Camacho & Alves-Souza (2018)	SLR	×	✓	2011–2017	4	✓	×	×	✓	×	×	×
Eirinaki et al. (2018)	Survey	×	✓	2000–2015	×	✓	×	×	×	×	×	×
DRSN	SLR	✓	✓	2011–2021	3	✓	✓	✓	✓	✓	✓	✓

The research articles are selected by study selection procedure which uses inclusion and exclusion criteria. It includes peer-reviewed articles. Moreover, this SLR only focuses on articles that are published from 2011 to 2021. Furthermore, it does not focus on articles not listed in the Journal Citation Report (JCR) list (Aloui, 2021). Likewise, the articles with no evaluation criteria or novel contribution are excluded. In case of some enhancement in the already published article, the only latest article will be considered.

Section 2 focuses on the literature review; Section 3 defines the research protocol; Section 4 answers the research questions; Section 5 shows the taxonomy based on the domain of DRSN and limitations of SLR; and Section 6 deals with general observation about SLR and future directions; Section 7 concludes the SLR.

## Literature Review

There are many surveys and a few SLR in the field of DRSN are discussed in this section, but some focuses on RS while other focuses on data mining rules on social media dataset. However, two surveys focus on both data mining and RS but they comprise outdated data and miss several aspects. The literature can be divided into three sections which are mentioned below.

### Data mining techniques on social network

This section discusses the surveys that gather data about the data mining techniques to store and manage data coming from social networks.

A survey article (Adedoyin-Olowe, Gaber & Stahl, 2013) focused on data mining techniques that are used in social network analysis. It focuses on pre-processing methodologies, data analysis, and data interpretation methodologies used in data mining on Web 2.0 technologies. This article limits its scope to data mining methods. It does not focus on the novel state-of-the-art methodologies in data mining. Also, it only shows the methodologies and tools used for data mining, but it does not work on where the mined data will be utilized afterward.

Furthermore, a survey (Injadat, Salo & Nassif, 2016) works on the methods of data mining on social media networks focuses on studies from 2003 to 2015. It selected 66 research articles, and results show that there are 19 techniques used for data mining in collaboration with social media content till that time. This study primarily focuses on nine research objectives in different domains, but the data mining methods are still raw and ambiguous in social media. This article focuses on data mining methods used in social media, its research areas, their incorporation with machine learning methodologies and comparison among data mining methods. It also mention the strengths, and weaknesses of using data mining on social media datasets. This article only focuses on publication venues along with chronological distribution. Rather, it focuses on machine learning-based ideas and methods to incorporate with social media. Furthermore, it does not focus on recommendation methods and data mining on social media data to make it efficient.

### RS methods on social network

This section discusses the 2 SLRs and 1 survey article that emphasizes on methodologies of RS while providing recommendations.

An SLR (Alrashidi et al., 2021) focuses on social networks and provides recommendations accordingly. It focuses on 32 articles from 2016 to 2020. Furthermore, it has used five digital libraries: Scopus, IEEEXplore, Springer, ScienceDirect, ACM, and Web of Science for data extraction. It also states that hybrid models in recommend systems result in high accuracy. It uses deep learning for emerging social recommend systems. This article only focuses on deep learning methodologies used for recommendation; however, this does not focus on data mining methodologies such as association, clustering, and anomaly analysis (Azzalini & Scarpa, 2012) explicitly for storing and managing large data produced by social websites. Moreover, potential issues or limitations are not discussed which to improve the system.

Another, SLR (Camacho & Alves-Souza, 2018) deals with review upon cold start problem (Lam et al., 2008) in which there is no information available in system for providing recommendations. This work focuses on studies from 2011 to 2017. This SLR classifies the possible solutions to eliminate social network issues in recommend systems. The scope is limited to dealing with the cold start problem rather than the complete domain of recommended systems. It deals with a specific type of article.

A survey article (Javed et al., 2021) focuses on content-based along with context-based RS by considering CBF, CF, and hybrid models. It considers the articles from 2000 to 2015. It shows the year-wise distribution of articles related to RS. It also shows the classification of methodologies used by RS. This article generally focuses on methods and techniques used by RS rather than focusing specifically on social networks and ways of mining the data.

For a large scale, social network data recommend systems are used. A survey based on large-scale social network data (Eirinaki et al., 2018) spotlights different recommendation methods. It also focuses on challenges faced by large-scale RS, such as data variety, volume, and volatility. Moreover, it discusses special issues articles in this domain. It emphasizes context-aware and simple item RS. Nevertheless, this does not focus on session-based RS using neural networks or any other data mining method that can be used to deal with huge data.

### Data mining rules along with RS

This section discusses the surveys that focus on both data mining and RS while providing recommendations.

The survey is concentrated on data mining methodologies in RS (Najafabadi, Mohamed & Mahrin, 2019). This article extracted 36 articles from IEEEXplore, ACM, Sage, and ScienceDirect. However, the scope of this article is limited to CF in RS. It does not examine other methodologies used in RS, such as content-based filtering and hybrid modeling. Likewise, an article emphasizes recommend systems using social media (Anandhan et al., 2018). Its scope is limited to two digital libraries and incorporates articles from 2011 to 2015. Furthermore, it only incorporates limited keywords for research such as “recommend systems,” “forums,” or “forum,” “social network” or “social networks,” or “web” or “social bookmarking” “blogs” or “blog .” It does not incorporate “data mining” or “social website.”

The articles (Adedoyin-Olowe, Gaber & Stahl, 2013; Injadat, Salo & Nassif, 2016) are surveying those centers on data mining methodologies in the social network. Whereas, Alrashidi et al. (2021) and Camacho & Alves-Souza (2018) are SLRs on RS, Eirinaki et al. (2018) is survey in the field of RS using social websites and Javed et al. (2021) is survey only discusses RS. Lastly, Najafabadi, Mohamed & Mahrin (2019) and Anandhan et al. (2018) focus on both data mining rules in RS using social media data, but they are survey studies. Table 2 shows the comparison of studies and methodologies they have adopted for review. We have not found an SLR in the domain of DRSN dealing with JCR-listed articles ranging from 2011 to 2021. The data mining and recommendation methodologies can be used in conjunction with each other that complement the results. Thus, there is a need for SLR that focuses on the domain of DRSN.

Table 2 shows that two studies work on the DRSN domain, but both are survey articles (Najafabadi, Mohamed & Mahrin, 2019; Anandhan et al., 2018). One of the articles only focuses on CF-based methodologies (Najafabadi, Mohamed & Mahrin, 2019), and other articles have not shown combined usage of data mining and RS on social networks (Anandhan et al., 2018). The articles (Adedoyin-Olowe, Gaber & Stahl, 2013; Injadat, Salo & Nassif, 2016) are centered on data mining. Alrashidi et al. (2021); Camacho & Alves-Souza (2018); Eirinaki et al. (2018) highlights RS based reviews and Javed et al. (2021) focuses on RS based methods but does not incorporate it on social networks using data mining rules.

There is a need for an SLR that deals with all the data mining and recommendation methodologies used in social networks without any biases. This SLR focuses on DRNS-based articles, thus capturing all the schemes used in this domain. It also extracts some necessary elements used for research such as dataset, evaluation metrics, techniques, and methods, along with considering the research trends by evaluating year-wise, country-wise, and publication venue-wise progress in the domain of DRSN thus, gathering all the details about articles.

Our proposed SLR is different from the reviews stated in Table 2 because we focus on different research questions that highlight on publication venues in which articles are published. Furthermore, we synthesized year-wise chronological distribution and geographical distribution of studies. Moreover, the proposed methodology is evaluated in terms of framework, scheme, method, model, algorithm, or application. Additionally, the domain-based categorization of the proposed solution is investigated. Likewise, an investigation is made on datasets and the size of datasets used by different studies. Similarly, analysis is made on evaluation criteria used for the validation of the proposed solution. Lastly, the identification of research gaps in DRSN is performed by considering its limitations. The next section discusses the research methodology that is being used to perfrom the SLR.

## Research Methodology

**Figure 1:**
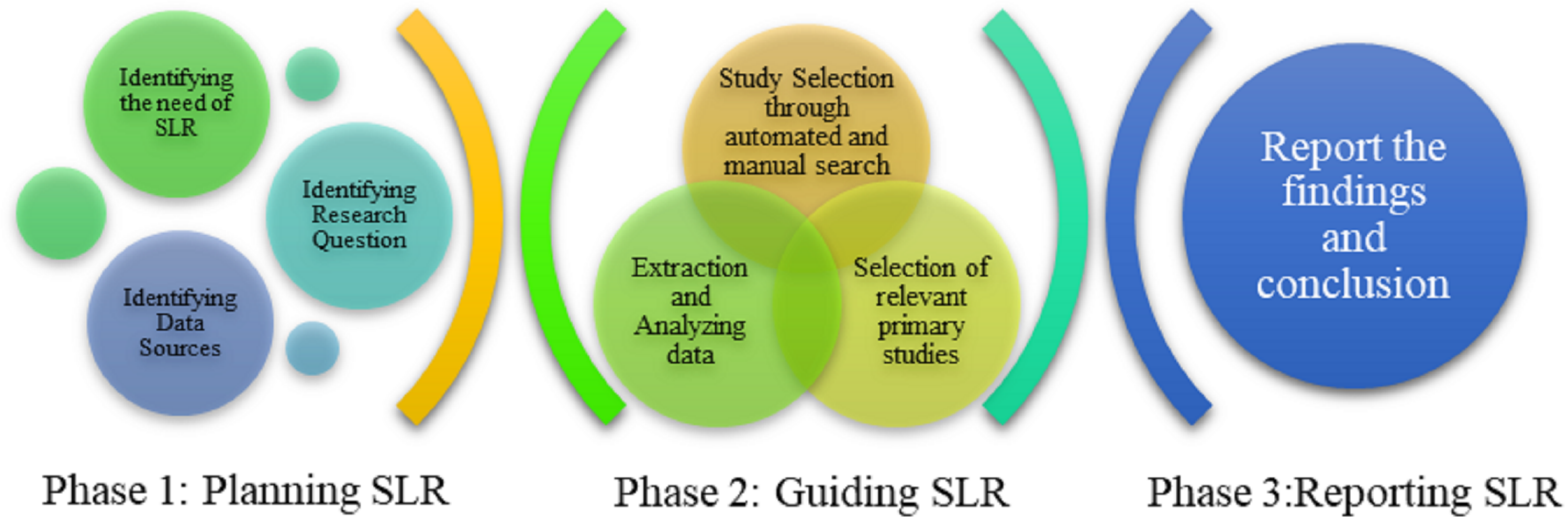
Phases of SLR (Kitchenham et al., 2009).

In this SLR, we highlight the procedure introduced by Kitchenham et al. (2009) which is based on principles of software engineering. This complete SLR is formulated and executed on Kitchenhem’s protocol. This protocol is mainly divided into three phases (Ajmal & Muzammil, 2019; Muzammil et al., 2021) which are shown in Fig. 1. The steps of SLR are discussed below.

### Planning SLR

At this phase, the structure of performing an SLR is planned by considering different characteristics of the review. This starts with the need of performing an SLR including the formation of research questions and identifying electronic databases from where articles are extracted.

#### Identifying the need of SLR

This SLR is needed because social media is a widely used platform at the current time and it produces a lot of content that needs to be mine for future predictions. No such work has been performed to date which works on the domain of DRSN explicitly and rigorously. The existing articles show that Adedoyin-Olowe, Gaber & Stahl (2013) and Injadat, Salo & Nassif (2016) are surveys that focus on data mining methodologies in a social network. Whereas, Alrashidi et al. (2021) and Camacho & Alves-Souza (2018) are SLRs, Eirinaki et al. (2018) is a survey in the field of recommended systems using social websites and Javed et al. (2021) is a survey that only focuses on RS in general. Lastly, Najafabadi, Mohamed & Mahrin (2019) and Anandhan et al. (2018) stress both data mining rules in RS using social media data, but it is a survey study with limited scope. Therefore, there is a need for an SLR that focuses on DRSN which includes articles from 2011 to 2021 and focuses on research objectives that find outs publication venues, year-wise distribution, country-wise distribution, publisher information, algorithms, schemes, data sets, size of data sets, evaluation metrics and limitations of existing articles focusing on DRSN.

#### Identifying data sources

The search strategy includes the identification of electronic databases (ED) that are explored to gather the studies on the topic. Table 3 shows the EDs that will be used in SLR. The databases that are used to perform this SLR are most relevant to the domain of DRNS as they are famous scientific EDs and helps to find the best results.

#### Identifying research questions

Research questions (RQ) are the most important part of SLR. RQ is used to perform research by answering them procedurally and systematically. Table 4 shows the RQ along with the motivation. These RQ will be addressed in the systematic literature review.

**Table 3:** Electronic databases.

**ID**	**Database**	**URL**
**ED1**	IEEE	https://ieeexplore.ieee.org
**ED2**	ACM	https://dl.acm.org
**ED3**	Springer	https://link.springer.com

**Table 4:** Research questions.

**ID**	**Research question**	**Motivation and objectives**
**RQ1**	What publication venues working on data mining in recommending systems upon social media websites? Explain their year-wise chronological distribution along with geographical distribution.	To find out
		• Publication venues for DRSN
		• Year-wise distribution of DRSN
		• Country-wise distribution in DRSN
		• Analysis based on information like publisher, country, proposed solution type and quality criteria score of selected studies in DRSN
**RQ2**	What are the different approaches of data mining along with recommendations adopted in social websites?	To find out the algorithms and schemes in which recommend systems are used with data mining.
**RQ3**	What kind of datasets metrics are adopted for conducting DRSN? Also, which methods are considered for validating the performance of DRSN?	To find out various detail in domain of DRSN
		• Dataset
		• Size of dataset
		• Evaluation metrics
**RQ4**	What are the limitations of using data mining in recommended systems upon social media websites?	To find out the limitations in the field of the social website based recommend systems to identify research gaps.

### Guiding the SLR

The most important component of an SLR is query formation. The query is generated by using different terms used in our topic and the conjunction of the “AND” and “OR” operators. Furthermore, to search data from the selected EDs, a research query is formulated to search data on the ED. The search query is the combination of keywords and their synonyms used in DRSN such as data mining, recommendation system, etc. While formulating search query different groups are made. Each group comprises a keyword and its synonyms which are joined by using “OR” in the search query and different groups are joined by using “AND”. The query components of DRSN are shown in Table 5, which collectively formulates a synthetic query.

**Table 5:** Query distribution. The asterisks (*) are due to a query that acts as a wildcard and will match any word or phrase.

	**Group 1**	**Group 2**	**Group 3**	**Group 4**
**Term 1**	Recommend*	Social	Media	Data mining
**Term 2**	Recommend* system		Forum	
**Term 3**	Expert system		Platform	
**Term 4**	Suggest*		Website*	

The following research query is formulated using Table 5 (Recommend* OR Recommend* System OR Expert System OR Suggest*) AND (Social) AND (Media OR Forum OR Platform OR Website*) AND (Data Mining) which can be applied on different ED specified in Table 4. The structure of queries writing is unique in different ED’s is shown in Table 6.

**Table 6:** Query structure on electronic databases.

**ID**	**Database**	**URL**	**Constrains**		
**ED1**	IEEE	(Recommend* OR Recommend* System OR Expert System OR Suggest*) AND (Social) AND (Media OR Forum OR Platform OR Website*) AND (Data Mining)	Journals		2011–2021
**ED2**	ACM	[[Title: recommend*] OR [Title: recommend* system] OR [Title: expert system] OR [Title: suggest*]] AND [Title: social] AND [[Title: media] OR [Title: forum] OR [Title: platform] OR [Title: website*]] AND [Title: data mining] AND [Publication Date: (01/01/2011 TO 12/31/2021)]	Journals		2011–2021
**ED3**	Springer	‘(Recommend* AND OR AND Recommend* AND System AND OR AND Expert AND System AND OR AND Suggest*) AND AND AND (Social) AND AND AND (Media AND OR AND Forum AND OR AND Platform AND OR AND Website*) AND AND AND (Data AND Mining)’	Article	Computer Sciences	2011–2021

#### Study selection criteria

There is a proper flow of selecting studies while performing SLR. It is necessary to pass all the phases to achieve better, meaningful, and quality studies. There are four steps in the study selection procedure which are illustrated in Fig. 1.

In identification phase the articles are first identified using a search query. Whereas, while screening the articles are scanned by title, abstract, and full text in sequence. The eligibility of selected studies are passed from inclusion and exclusion criteria. Lastly, final inclusion depends upon quality assessment criteria and duplication removal.

#### Identification and selection of primary studies

The primary studies are identified based on the search query, screened by title, abstract, and full text analysis. The eligibility of selected articles are checked by considering inclusion and exclusion criteria determined in Tables 7 and 8.

**Table 7:** Inclusion criteria.

**ID**	**Inclusion criteria**
IC1	Articles should be peer reviewed.
IC2	Articles should be in domain of DRSN
IC3	Articles should target the research questions.
IC4	Articles whose full text is available
IC5	Articles published in the time period of 2011 and 2021.
IC6	Articles presenting novel research contributions in domain of DRSN
IC7	Articles having no validation their novelty and total relevance can be used
IC8	In case of some enhancement in already published article related to DRSN, the latest article will be considered.
IC9	The article is JCR listed journal article in field of DRSN

**Table 8:** Exclusion criteria.

**ID**	**Exclusion criteria**
EC1	Articles other than English
EC2	Articles that does not discusses results and experiments.
EC3	Articles related to search article recommendation.
EC4	The article other than journal article.
EC5	The articles other than the domain of DRSN should not be discussed.

Studies that fulfill the eligibility phase are scanned for quality assessment criteria to include the final articles. The quality assessment criteria are shown in Table 9.

**Table 9:** Quality assessment criteria.

**ID**	**Quality criteria**	**Score**
QAC1	The goals of articles along with objectives should be clear.	1
QAC2	The article should explain its results and experiments properly in detail.	1
QAC3	The limitations should be stated explicitly in the article.	1
QAC4	The article should have checks for novelty along with validation of proposed solution.	1

#### Extraction of data

For data extraction according to the requirements and RQs. The data is obtained from selected studies. Table 10 helps to excerpt data from articles.

**Table 10:** Data extraction form.

**Article name**	**Description**
**Citation information**	It includes name of publisher, journal, year of publication, country of first author, and year of publication
**Purpose**	This gathers the purpose or need of the study.
**Publication venue**	It includes name of the publication venue or journal.
**Publisher**	This state the publisher in which the journal is referenced.
**Year**	It includes the year in which article is published.
**Country**	This states the country of article’s first author
**Method/Framework/** **Technique**	This will state the type of proposed methodology suggested by the authors
**Domain**	It states the community that can get benefit from the study.
**Algorithm**	It shows the algorithm used by studies as their proposed solution.
**Datasets**	This includes the dataset that studies used to per from their experiments.
**Datasets size**	This includes the size of data set that studies used to per from their experiments.
**Evaluation metrices**	This demonstrates the evaluation methods that are used by studies to perform validation of their proposed system.
**Research gaps**	It identifies the limitations of the studies that will leads to the research gaps for new studies.
**QAC1, QAC2, QAC3, QAC4**	This evaluates the articles score on the basis of quality assessment criteria shown in Table 9.

### Reporting SLR

**Figure 2:**
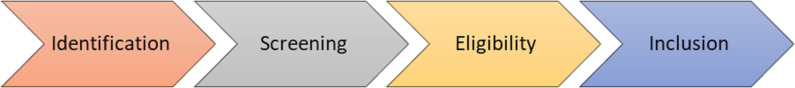
Phases of study selection.

**Figure 3:**
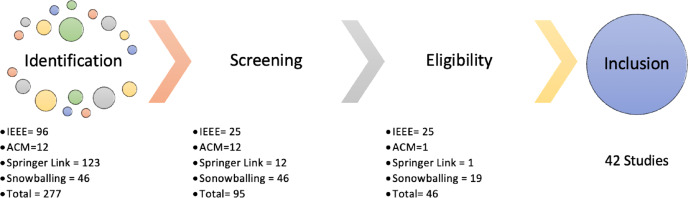
Phases of study selection.

In the current era, DRSN is an active field because of the immense use of social media. Our SLR identifies the articles relevant to DRSN from different electronic databases. A total of 200+ articles are extracted in the initial phase from 2011 to 2021. The articles are then filtered out by the study selection procedure shown in Fig. 2. Figure 3 shows the extraction of articles based on the steps shown in Fig. 2. The identification phases comprise all the studies that are extracted from different databases. We have only applied three EDs explained in Table 3. Initially, we had 277 articles in the identification phase. In the screening phase, the articles are skimmed based on title and abstract which results in 95 articles. In the third step, eligibility is ensured by considering inclusion, and exclusion criteria and a total of 46 articles are extracted. Finally, studies are selected using quality criteria which are 42 in number.

The quality criteria shown in Table 8 are checked for each article. Four quality criteria standards should qualify to be part of SLR. It has total four marks, one for each QAC. Table 11 shows the criteria of scores. The count of eligible articles concerning the score is shown in Table 12.

**Table 11:** QAC score mapping.

**QAC score**	**Mapping**
1	Completely available
0.75	Partially available
0.5	Neutral
0.25	Very less available
0	Not available

**Table 12:** Count of articles with respect to quality assessment score.

**Total Score = 4**					
**Publication score**	+3.5	+3	+2.5	+2	+1.5
**Count**	5	14	10	9	4

**Figure 4:**
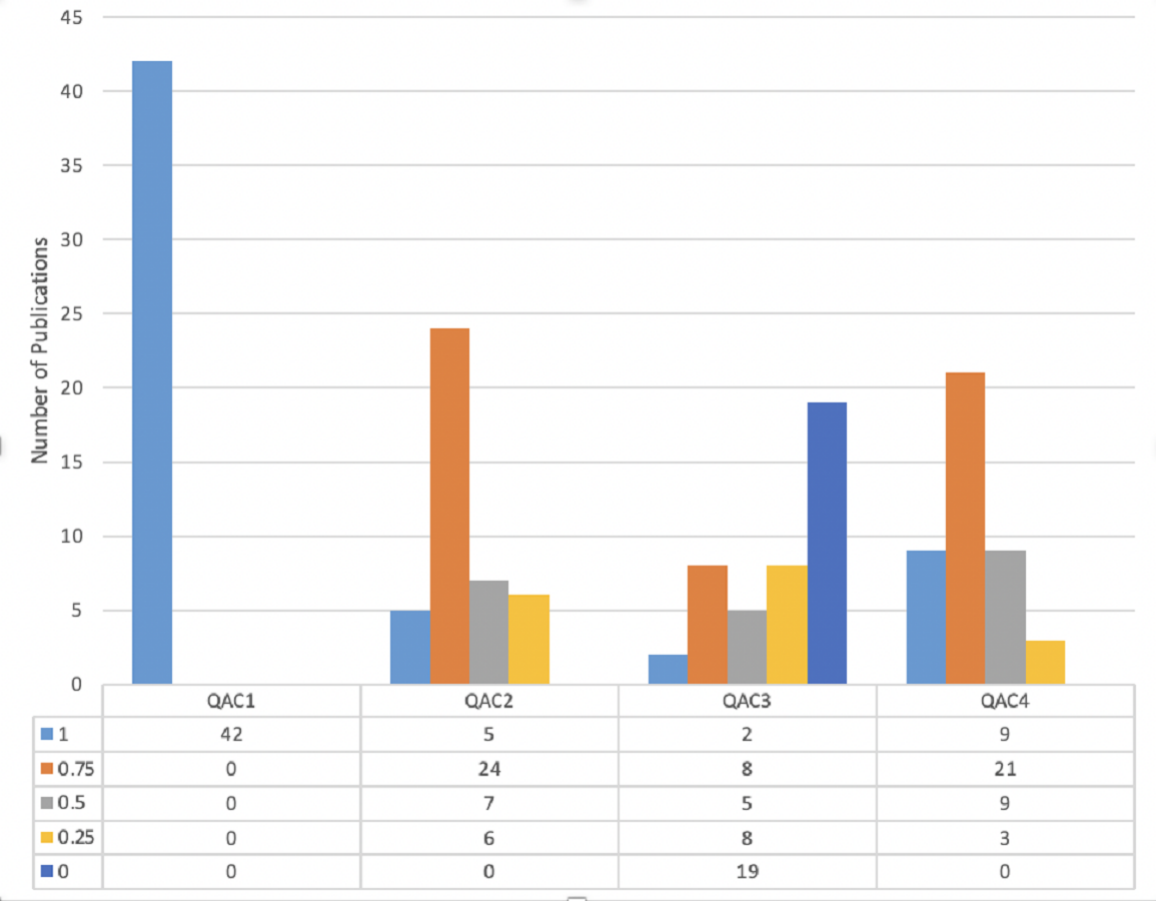
Score of articles on the basis of quality criteria.

The articles with a quality score of more than 1.5 out of four are added to this SLR. Figure 4 shows the quality ratio of articles related to each QAC, which shows that all articles have declared their goals and objectives of studies. In QAC2, 12% articles have appropriately shown their experiments and results, given 1 score, whereas, 57% articles have received 0.75 scores, 17% comprises of 0.5 scores, and 14% acquire 0.25 score. On the other hand, in QAC3 only 55% of articles explained their limitations, and 45% have not discussed it at all. Lastly, in QAC4 the novelty of work and its validation is explained by 22%, 50%, 21%, 7%, and 0% articles having 1, 0.75. 0.5, 0.25, and 0 score accordingly. So, a total of 42 articles were selected for performing SLR.

## Results and Discussion

This section discusses the selected 42 articles based on the proposed research questions.

### RQ 1: What publication venues working on data mining in recommended systems upon social media websites? Explain their year-wise chronological distribution along with geographical distribution.

**Figure 5:**
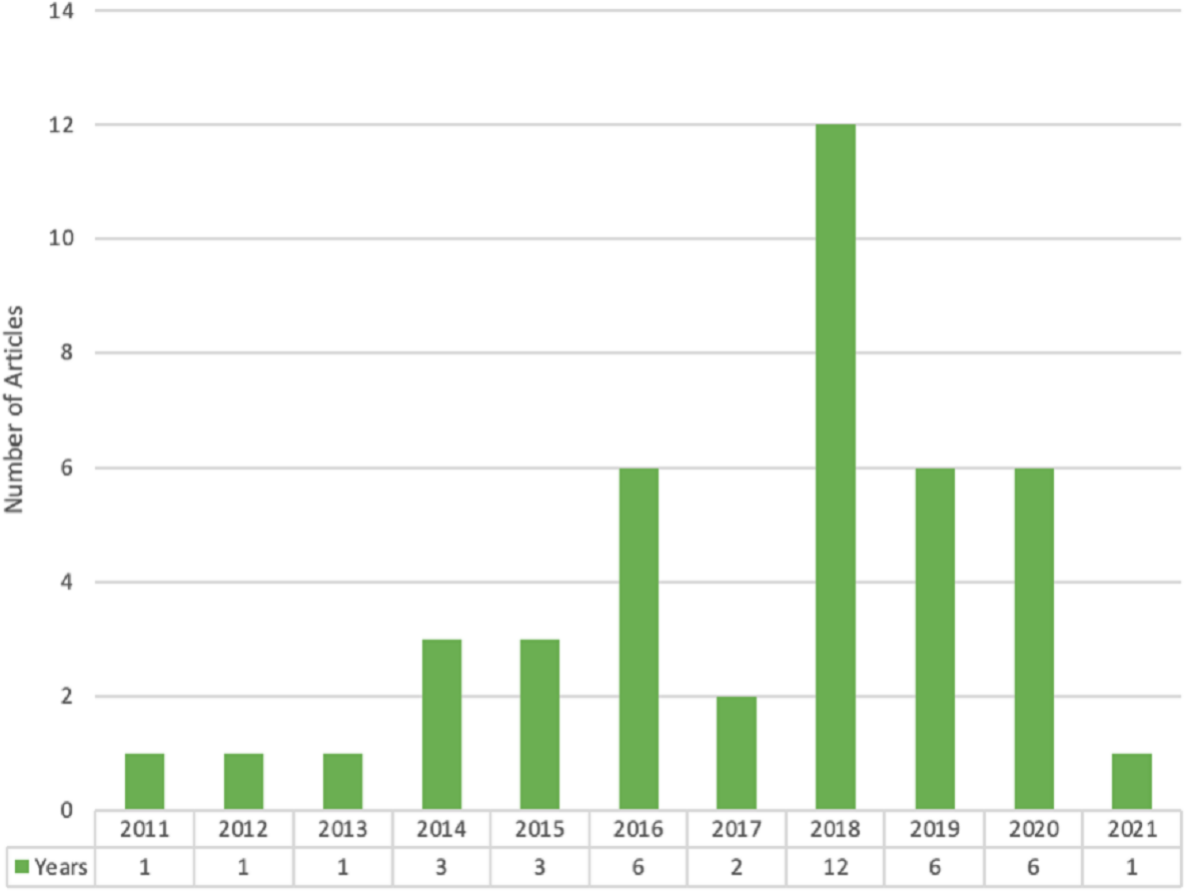
Year wise chronological distribution.

The data mining methodology is essential to mine data to provide recommendations. The data produced by social media websites are considered big data that needs to be stored and mined properly using data mining methods to get suitable recommendations. The 42 selected studies are classified concerning publication venue, publisher, country, and proposed solution. Figure 5 shows the chronological year-wise distribution of selected articles. It depicts those nine articles published in 2018, which is the highest number of articles in any year, and the second-highest is eight articles published in 2020.

**Figure 6:**
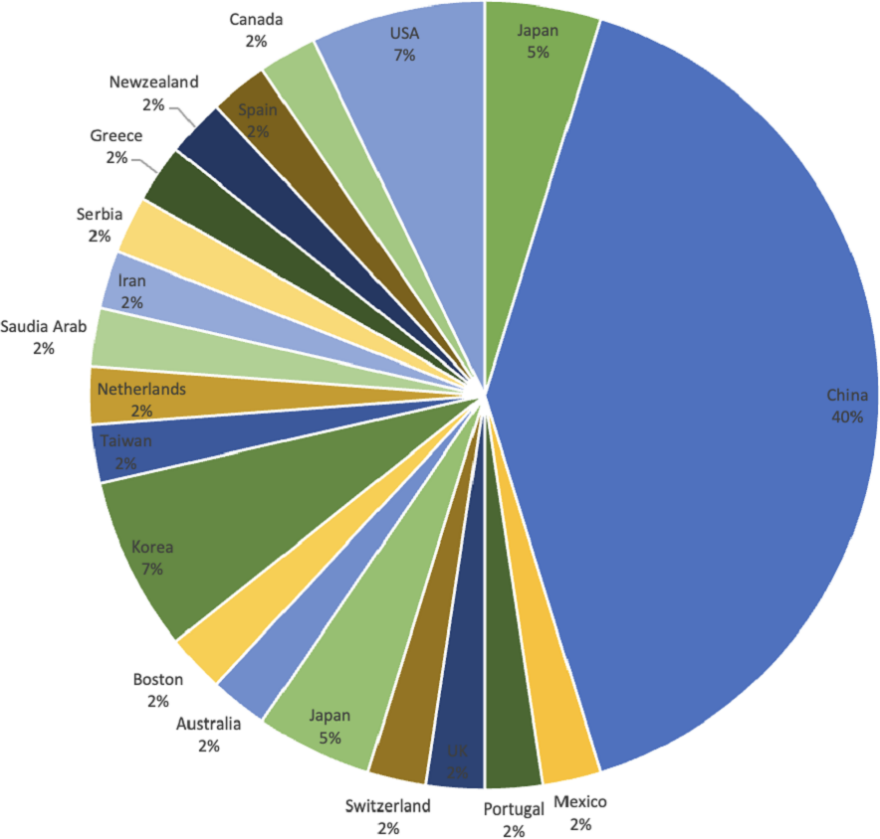
Geographical distribution.

Figure 6 displays the geographical distribution according to the first author of the journal. It is observed that 17 out of 42 articles are published in China, demonstrating that 40% contribution in the domain of DRSN is by Chinese authors.

**Figure 7:**
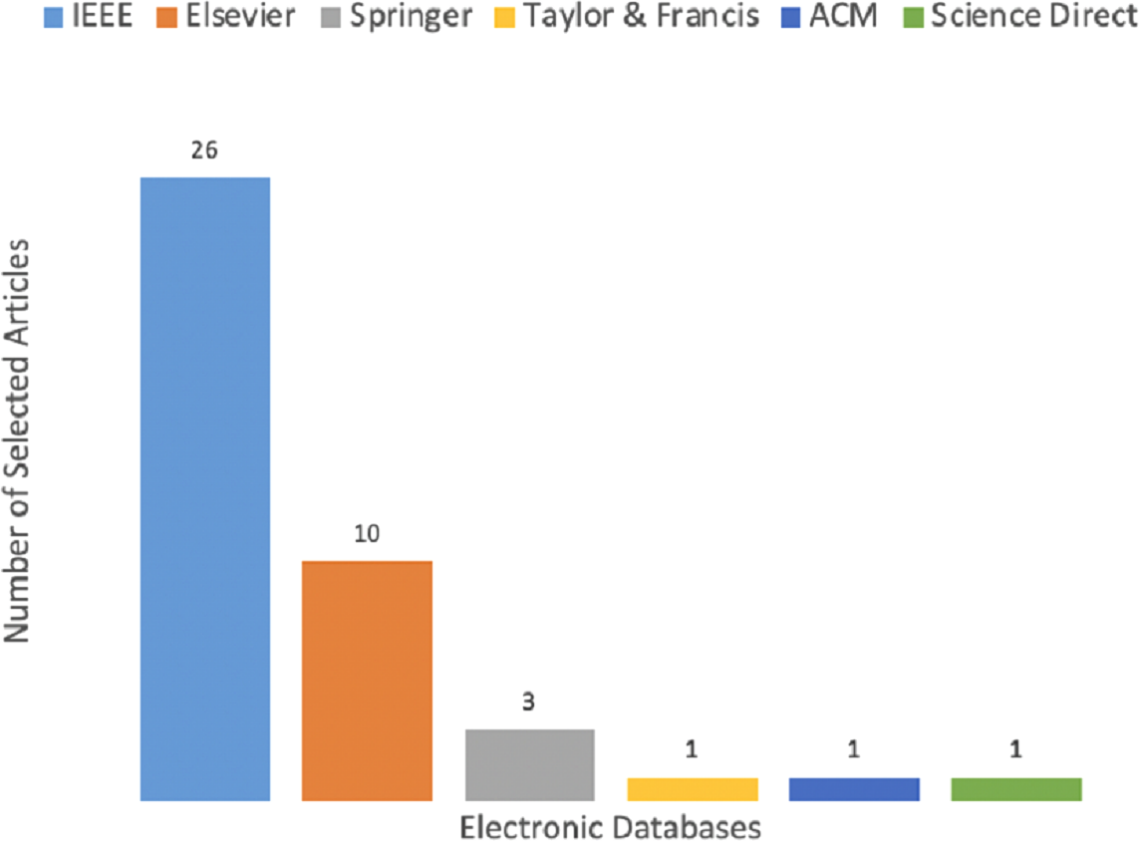
Publication venues wise distribution.

Tables A1 in the appendix shows the categorization of all selected articles according to the year they are published. It comprises publication venues, publisher, year of publication, geographical location of the first author, type of proposed methodology, and quality assessment score. The QAC is evaluated based on criteria shown in Table 9. From Table A1, it is synthesized that 26 articles are published in IEEE, ten are published in Elsevier, three are extracted from Springer, one from ACM, and Science Direct, which is demonstrated in Fig. 7. The IEEE, ACM, and Springer databases are used during study selection process however, others are identified using the snowballing process of finding articles.

**Figure 8:**
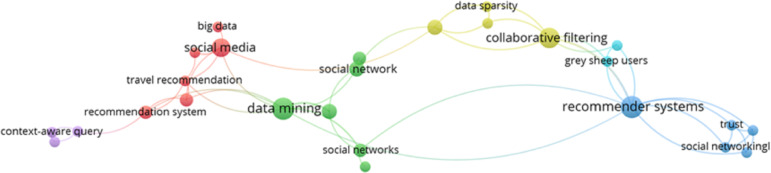
Research trends of DRSN.

Moreover, the research trend of DRSN can be evaluated in Fig. 8, which shows that data mining, RS, and social networks are related to each other. It also shows some of the terms related to the domain. This figure is generated to check the research trend of selected articles based on the most frequently occurring words in their abstracts.

### RQ 2: What are the different approaches to data mining or recommendation adopted in the recommendation of social websites?

**Figure 9:**
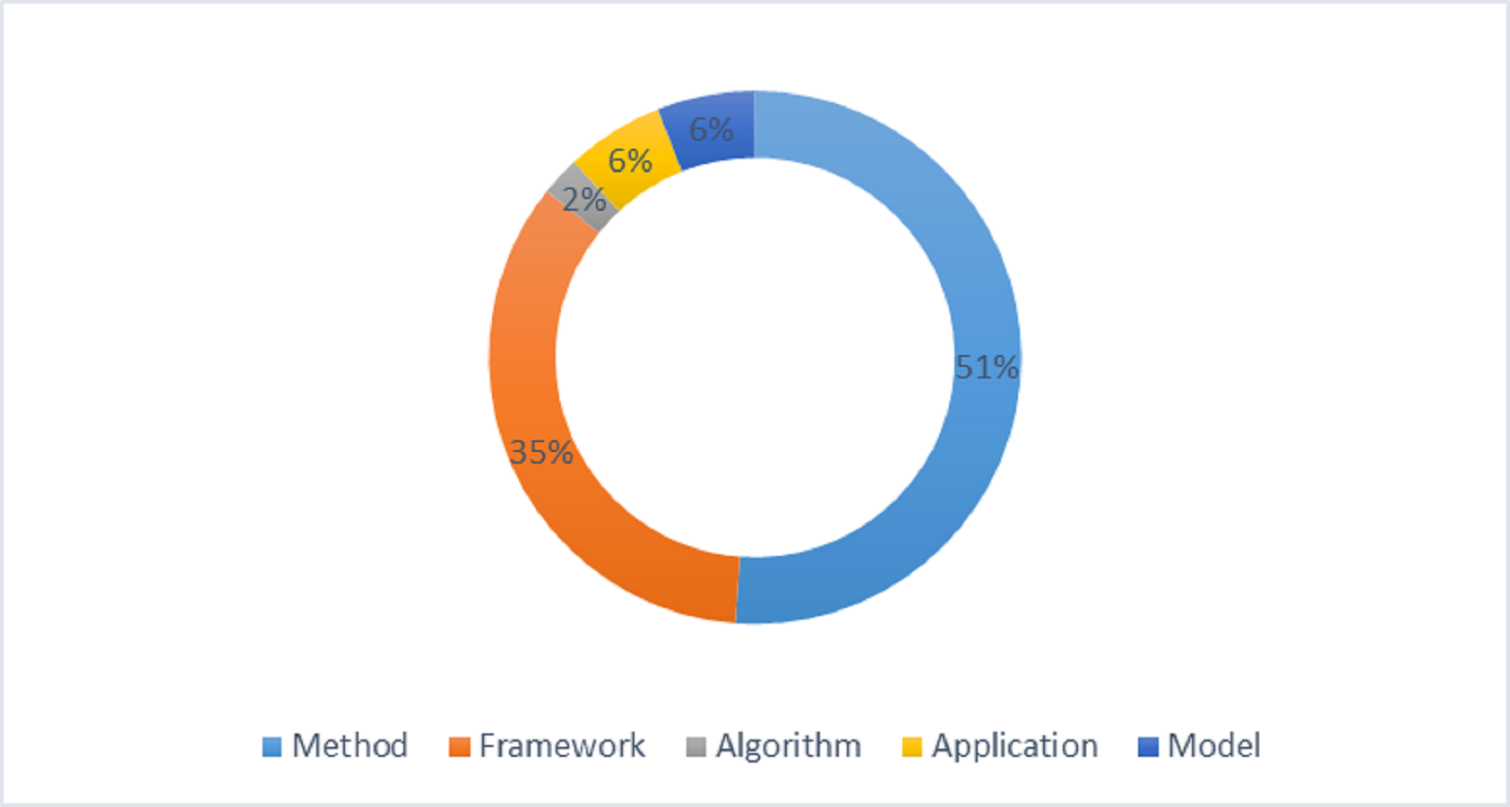
Percentage distribution of proposed solution.

The proposed solution can be an algorithm, method, framework, model, or application. It is analyzed that 35% solutions are frameworks, 51% are methods, 6% are applications, 6% are models, and 2% are algorithms. Figure 9 expresses the percentage distribution of the proposed solution.

**Figure 10:**
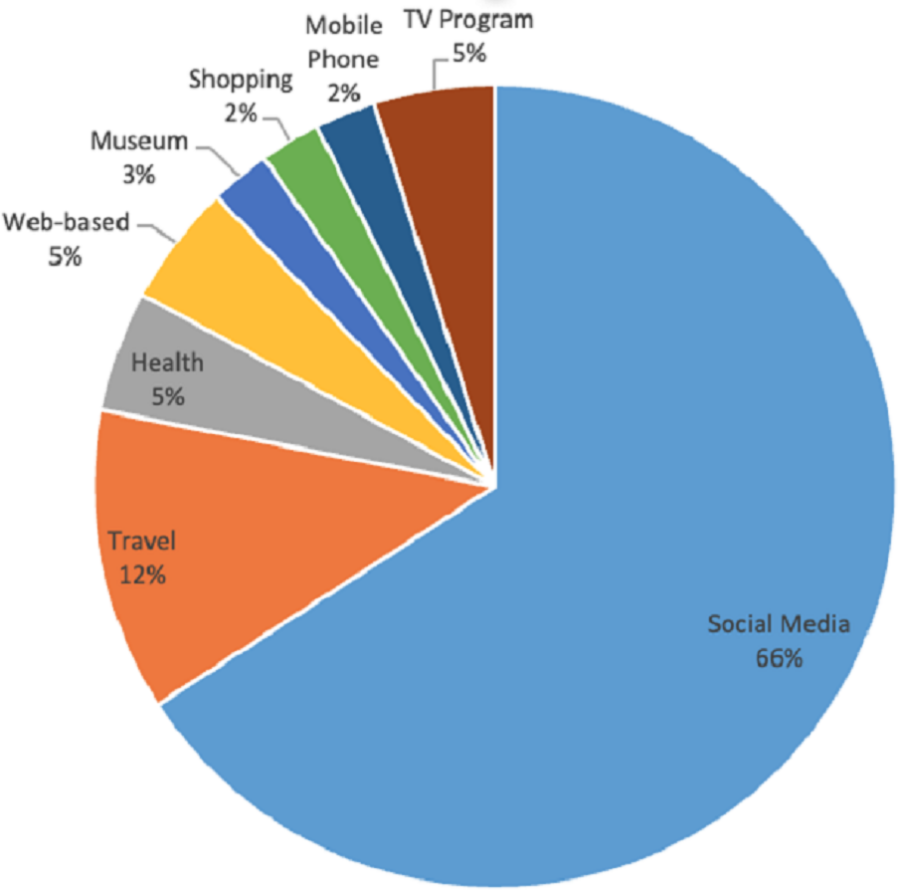
Domain-wise distribution.

Furthermore, it analyzed that the proposed solution can work on social media generically or use social media data to apply it to specific domains. Social media is considered by 27 studies. On the other hand, five articles uses it for travel purposes (Fang et al., 2014; Wang et al., 2014; Xu, Chen & Chen, 2015; Zhao, Qian & Xie, 2016; Jamiy et al., 2015), two articles use it in health (Zhao et al., 2018; Zhang et al., 2019), two article uses it as a web based proposed solution (Jiang et al., 2016; Alduaiji, Datta & Li, 2018), two articles uses for mobile phone based solutions (Zanda, Eibe & Menasalvas, 2012; Majid et al., 2013), a single article focused on museum (Qin et al., 2018), one article deals with shop type recommendations (Moro, Rita & Vala, 2016), and an article deals with stock data (Zhou et al., 2019). The distribution of domains in DRSN is shown in Fig. 10.

The DRSN mainly focuses on data mining and recommendation techniques. The data mining methodologies includes bayesian network (Min & Cho, 2010), clustering (Zanda, Eibe & Menasalvas, 2012; Yang, Qu & Cudré-Mauroux, 2018; Qin et al., 2018; Milovanović et al., 2019), k-NN (Majid et al., 2013; Adeniyi, Wei & Yongquan, 2016; Nguyen & Cho, 2020; Xu, 2018), topic season matrix mining (Jiang et al., 2016); support vector machines, Moro, Rita & Vala (2016) and Torres-Ruiz et al. (2020), nave based (Torres-Ruiz et al., 2020), decision trees (Torres-Ruiz et al., 2020), single vector decomposition (Qin et al., 2018), association mining (Sang, Yan & Xu, 2018; Dhelim et al., 2020), regression (Zhou et al., 2019), semantic analysis (Sun, Fang & Wang, 2018), and neural networks (Zhou et al., 2019). Whereas RS is divided into three main state-of-the-art categories such as CBF, CF, and hybrid mode. The CBF comprises of some algorithms named term frequency-inverse domain frequency (Majid et al., 2013; Jiang et al., 2016; Wen et al., 2017; Psyllidis, Yang & Bozzon, 2018) topic modeling (Wang et al., 2014; Lwowski, Rad & Choo, 2018), latent Dirichlet allocation (Wang et al., 2014; Fang et al., 2014; Pyo & Kim, 2014; Jiang et al., 2015; Xu, Chen & Chen, 2015; Shi et al., 2017; Sang, Yan & Xu, 2018; Cui et al., 2018; Psyllidis, Yang & Bozzon, 2018; Nguyen & Cho, 2020; Ge et al., 2020) feature extraction (Yu et al., 2016), word2vec (Zhao et al., 2018), and natural language processing (Psyllidis, Yang & Bozzon, 2018). On the other hand, CF uses user-item matrix (Pyo & Kim, 2014; Jiang et al., 2015; Moro, Rita & Vala, 2016; Iqbal et al., 2019; Manca, Boratto & Carta, 2018; Zhang et al., 2019; Ju, Wang & Xu, 2019; Margaris, Vassilakis & Spiliotopoulos, 2020; Shahbaznezhad, Dolan & Rashidirad, 2021), friend-matching graph (Wang et al., 2014), social network analysis (Wu et al., 2015; Wu et al., 2019), matrix factorization (Zhao, Qian & Xie, 2016; Yu et al., 2016; Zhao et al., 2018; Xu, 2018), classification (Yang & Jiang, 2018), and graph theory (Alduaiji, Datta & Li, 2018; Ahmadian et al., 2020). Therefore, in DRSN, it is observed that data mining and recommendation methods are used interchangeably to provide better results. Moreover, the hybrid mode is also being used by different studies in terms of algorithm concatenation. Table 13 shows the selected studies’ methods, schemes, or algorithms. It is observed that most of the studies use CF for providing recommendations to users and cluster data for mining based on using the same method. Around nine out of 42 studies use CF in the domain of DRSN. Other schemes are not used this frequently.

**Table 13:** Schemes and algorithms used in domain of DRSN.

**Ref.**	**Schemes and algorithms**
Min & Cho (2010)	Bayesian network
Zanda, Eibe & Menasalvas (2012)	Latent, Clustering
Majid et al. (2013)	K-NN, Collaborative Filtering, Similarities among users, Location based semantic filtering using Term Frequency-Inverse Domain Frequency
Fang et al. (2014)	Generalized Expectation Maximization for Hypergraph Regularized Topic Model, Topic-sensitive Influence Ranking
Pyo & Kim (2014)	Asymmetric Dirichlet prior, Latent Dirichlet Allocation, Collaborative Filtering
Wang et al. (2014)	Topic modeling using Latent Dirichlet Allocation, Friend-matching graph
Jiang et al. (2015)	Author topic modeling, Collaborative Filtering, Ranking
Xu, Chen & Chen (2015)	Probabilistic Latent Sementic Analysis
Wu et al. (2015)	Social Network, Decision Making Process
Adeniyi, Wei & Yongquan (2016)	K-NN Classification
Jiang et al. (2016)	Term Frequency-Inverse Domain Frequency, Natural Languge Processing, Topic’s Season Matrix Mining, Images Mining, Route Mining (Similar User Mining)
Zhao, Qian & Xie (2016)	Probabilistic matrix factorization + fuse user personal interest, interpersonal interest similarity, interpersonal rating behavior similarity, and interpersonal rating behavior diffusion, into matrix factorization
Moro, Rita & Vala (2016)	Collaborative filtering
Yu et al. (2016)	Novel bias learning matrix factorization, Feature extraction on location and commercial shops
Moro, Rita & Vala (2016)	Support vector machines
Wen et al. (2017)	Term Frequency-Inverse Domain Frequency + Greedy scoring using multidimensional index
Shi et al. (2017)	Latent Dirichlet Allocation, Automated event filerting
Yang & Jiang (2018)	Binary classification model for thread recommendation. Node-Based Features: Path-Based Features:Thread–thread relationship+User–user relationship.
Zhao et al. (2018)	Matrix factorization, Word2vec
Sang, Yan & Xu (2018)	Session-based user modeling (LDA) and dynamic association mining
Lwowski, Rad & Choo (2018)	Topic modeling, Emotion analysis, NP-Hard, Emotion Distribution Event Detection
Psyllidis, Yang & Bozzon (2018)	Natural Language Processing +document-feature matrix + Term Frequency-Inverse Domain Frequency, Latent Dirichlet Allocation, User modeling (through SocialGlassa), Clustering
Xu (2018)	Matrix Factorization, KNN
Alduaiji, Datta & Li (2018)	Graph Partition
Manca, Boratto & Carta (2018)	Tag-based Collaborative Filtering
Sun, Fang & Wang (2018)	Semantic Analysis
Yang, Qu & Cudré-Mauroux (2018)	Cluster-wise obfuscation function learning+Probabilistic historical data obfuscation, Personalized activity-wise obfuscation function learning, Probabilistic online activity obfuscation
Qin et al. (2018)	Clustering + Singular Value Decomposition
Cui et al. (2018)	Latent Friends mining + K-means clustering algorithm + all reviews from a user and all tags from their corresponding items algorithm, weighted local random walk with restart
Milovanović et al. (2019)	Tabu Search Clusteriing
Zhou et al. (2019)	DNN-based learning model with a Soft- Max classifier, CNN-based framework
Zhang et al. (2019)	Collaborative Filtering, User-item matrix
Wu et al. (2019)	Co-tags relation, user social realtion + user based Collaborative Filtering
Ju, Wang & Xu (2019)	Collaborative Filtering
Iqbal et al. (2019)	Additive and Multiplicative Models for both user- and item-based versions.
Torres-Ruiz et al. (2020)	Pattern Matching (Decision Trees, Single Vector Machine, Nave Based), Semantic Analysis by Vectors (IoT, Big Data, and Mobile Augmented Reality)
Dhelim et al. (2020)	Interest mining, item mapping, discover metapaths, Topic-Item based Associations
Nguyen & Cho (2020)	Expectation Maximization algorithm, user specific preference, latent group preference
Ge et al. (2020)	KKT+ Latent Dirichlet Allocation
Ahmadian et al. (2020)	Link Prediction, Graph Theory
Margaris, Vassilakis & Spiliotopoulos (2020)	Collaborative filtering
Shahbaznezhad, Dolan & Rashidirad (2021)	Collaborative filtering

### RQ 3: What kind of datasets metrics are adopted for conducting DRSN? Also, which methods are considered for validating the performance of DRSN?

Datasets are an important part of any research process. All the datasets used by selected articles in the domain of DRSN are shown in Table 14. It is evaluated that Twitter, Facebook, Flicker, and Foursquare are used by eight, six, six, and four studies, respectively. Three or fewer studies use the remaining datasets. Through all of available social networks, Twitter user population shows the consistent growth trends it provides to a platform for people to connect, communicate and exchange their experiences with each other. However, to perform a through analysis on social media twitter is widely adopted by many researchers. One phenomena behind this can be the reason as twitter provides a broad range of users’ activity data points and an easy access to this data. It is observed that five of 42 articles uses the real-time data set (Min & Cho, 2010; Wang et al., 2014; Wu et al., 2015; Xu, 2018; Ju, Wang & Xu, 2019). All the other articles used offline mode datasets. Table 14 also discusses the size of the dataset used by different studies.

**Table 14:** Datasets and evaluation metrics used in domain of DRSN.

**Ref.**	**Datasets**	**Size of the dataset**	**Evaluation metrics**
Min & Cho (2010)	Real time data from application	Not Mentioned	Accuracy
Zanda, Eibe & Menasalvas (2012)	Facebook	1379 posts	Time cost
Majid et al. (2013)	Flicker	736,383 geotagged images	Precision Benefit Ratio, Mean Average Precision
Fang et al. (2014)	Flicker	556,942 photos, 8,479 tags	Normalized Discounted Cumulative Gain Mean Average Precision
Wang et al. (2014)	Real time data from application	8 volunteers	Similarity threshold Recall Precision Energy consumption
Pyo & Kim (2014)	TNmS Korea Inc	4,313 TV programs 20 TV channels	Precision Recall Log-likelihood
Jiang et al. (2015)	Flicker	7 million photos	Mean Average Precision
Xu, Chen & Chen (2015)	Flicker	Not Mentioned	Mean Average Precision
Wu et al. (2015)	Real time data from application	30 users	User Feedback
Adeniyi, Wei & Yongquan (2016)	Feeds of different news websites	Not Mentioned	Euclidian distance
Jiang et al. (2016)	IgoUgo.com	24,008 travelogues of 864 locations	Mean Average Precision Top
Zhao, Qian & Xie (2016)	Yelp Douban Movie	3,468,485 ratings from 11,668 users who rated a total of 59,704 movies	Root-Mean-Square Deviation Mean Absolute Error
Moro, Rita & Vala (2016)	Facebook	790 posts	Mean absolute percentage error
Yu et al. (2016)	Dianping Location-Based Services (LBS) provider (Baidu LBS 2000).	Not Mentioned	Mean Absolute Error Normalized Discounted Cumulative Gain
Moro, Rita & Vala (2016)	Facebook	790 posts	Lifetime Post Consumers
Wen et al. (2017)	Flicker TripAdvisor	96 volunteers	Precision Top Performance Relative ratio
Shi et al. (2017)	Twitter	126,995 posts and 6,589 users	Similarity Analysis
Yang & Jiang (2018)	MedHelp	701 threads containing 3,759 messages.	Precision Recall F1 Score
Zhao et al. (2018)	Yelp	8,629 users, 96,974 items, and 300,847 ratings	Root-Mean-Square Deviation Mean Absolute Error
Sang, Yan & Xu (2018)	YouTube Twitter	2,522 users and 2,859 videos	Precision Recall F1 Score
Lwowski, Rad & Choo (2018)	Twitter	1,025,000 tweets	Prediction
Psyllidis, Yang & Bozzon (2018)	Foursquare Twitter	Not Mentioned	F-measure F1 score
Xu (2018)	MovieLens Book-crossing Real world	1,682 movies, 943 users and 100,000 ratings, 271,379 books and 1,149,780 ratings, 4,377,223 ratings, 384,374 products, 689,922 users	Precision Recall F-measure Root Mean Square Error Hamming Distance
Alduaiji, Datta & Li (2018)	Twitter Facebook Amazon	Not Mentioned	F1 Score Normalized F1
Manca, Boratto & Carta (2018)	Delicious dataset	1,867 users; 69,226 URLs; 53,388 tags; 7,668 bi-directional user relations; 437,593 tag assignments (i.e., tuples [user, tag, URL]); 104,799 bookmarks (i.e., distinct pairs [user, URL]).	Precision, % of satisfied users, Novelty
Sun, Fang & Wang (2018)	Guba	500 million stock posts, 1.5 million+ users	Correlation Agreement Cumulative Return Rate
Yang, Qu & Cudré-Mauroux (2018)	Foursquare	N/A	Mean Average Precision 1 Area Under Curve
Qin et al. (2018)	Flicker MovieLens MovieLens (1M)	2M images, 21 million ratings of 31,178 movies, 144,533 users. users.	Precision Recall Normalized Discounted Cumulative Gain
Cui et al. (2018)	Epinions Foursquare	6,855 users, 4,828 items, 115,505 ratings, 184,686 reviews	Precision Recall Normalized Discounted Cumulative Gain Mean Reciprocal Rank
Milovanović et al. (2019)	Survey on mobile applications	Not Mentioned	Cluster Analysis
Zhou et al. (2019)	HaoDaiFu online	11,000 physicians	Loss Comparison Accuracy PR curve F1 Score
Zhang et al. (2019)	Shareteches	738 users, 8,890 videos, 7,439 playing history records	Precision Recall F1 Score
Wu et al. (2019)	last.fm delicious	3,759 users, 122,431 services, 65,334 tags	Precision Recall Normalized Discounted Cumulative Gain Mean Reciprocal Rank
Ju, Wang & Xu (2019)	Real world dataset of sina microblog	7,209 users, 92,615 friends relationship, 72,059 user activity	Mean Absolute Error
Iqbal et al. (2019)	LDOS-CoMoDa DePaulMovie (Movie-based)	365 users, 4,460 movies and 4,998 rating records	Root-Mean-Square Deviation F1 Score
Torres-Ruiz et al. (2020)	Foursquare Facebook, Twitter, comments of Web sites referent to various museums in the Mexico City. beacon sensors in rooms of meseum and find geo location	Not Mentioned	Accuracy App testing
Dhelim et al. (2020)	Newsfulness (BBC, CNN, Aljazeera, France24, RussiaToday, Reuters, The Guardian, The NY Times)	2,228 users, 2,873 articles, 6,230 items	Precision Recall F1 Score
Nguyen & Cho (2020)	Gowalla Twitter llastfm redditS	Not Mentioned	Recall Area Under Curve Avg Rank Normalized Discounted Cumulative Gain
Ge et al. (2020)	Twitter	12,6995 posts and 6,589 users	No. of topics Effectiveness
Ahmadian et al. (2020)	Epinions Flixster FilmTrust	61,276 users and 141,809 items 35,497 ratings.	Mean Absolute Error Root-Mean Squared Error Precision Recall Diversity Novelty
Margaris, Vassilakis & Spiliotopoulos (2020)	Amazon YELP	Not Mentioned	Accuracy User Feedback overhead induction by computation
Shahbaznezhad, Dolan & Rashidirad (2021)	Facebook Instagram	1,038 social media posts, 1,336,741 likes, and 95,996 comments	Regression analysis

Several methodologies have been adopted to analyze and validate the research in various studies using evaluation parameters. It is part of experimentation, used to justify the research work using qualitative and quantitative experimentation. It is evaluated that one article uses a qualitative way of evaluating results (Wu et al., 2015) by using feedback from the user. At the same time, all other studies use the quantitative method using some evaluation metrics to evaluate the validity of studies. The evaluation metrics used in the domain of DRSN are shown in Table 14. A total of 12 studies use precision and recall for evaluation independently. As precision and recall provides true insights about the recommendations, which cannot be provided by accuracy. The true positives (TP), true negatives (TN), false positive (FP) and false negative (FN) are very crucial for providing recommendations, specially in health care. To detect TP, TN, FP and FN in efficient way, precision and recall are well suited. For this reason, it is used by most of the studies. Whereas the F1 score is used by eight articles, the root mean square error (RMSE) and mean absolute error (MAE) are used by five studies. Lastly, mean average precision (MAP) is used in four studies. Less than four articles use the remaining evaluation metrics.

**Figure 11:**
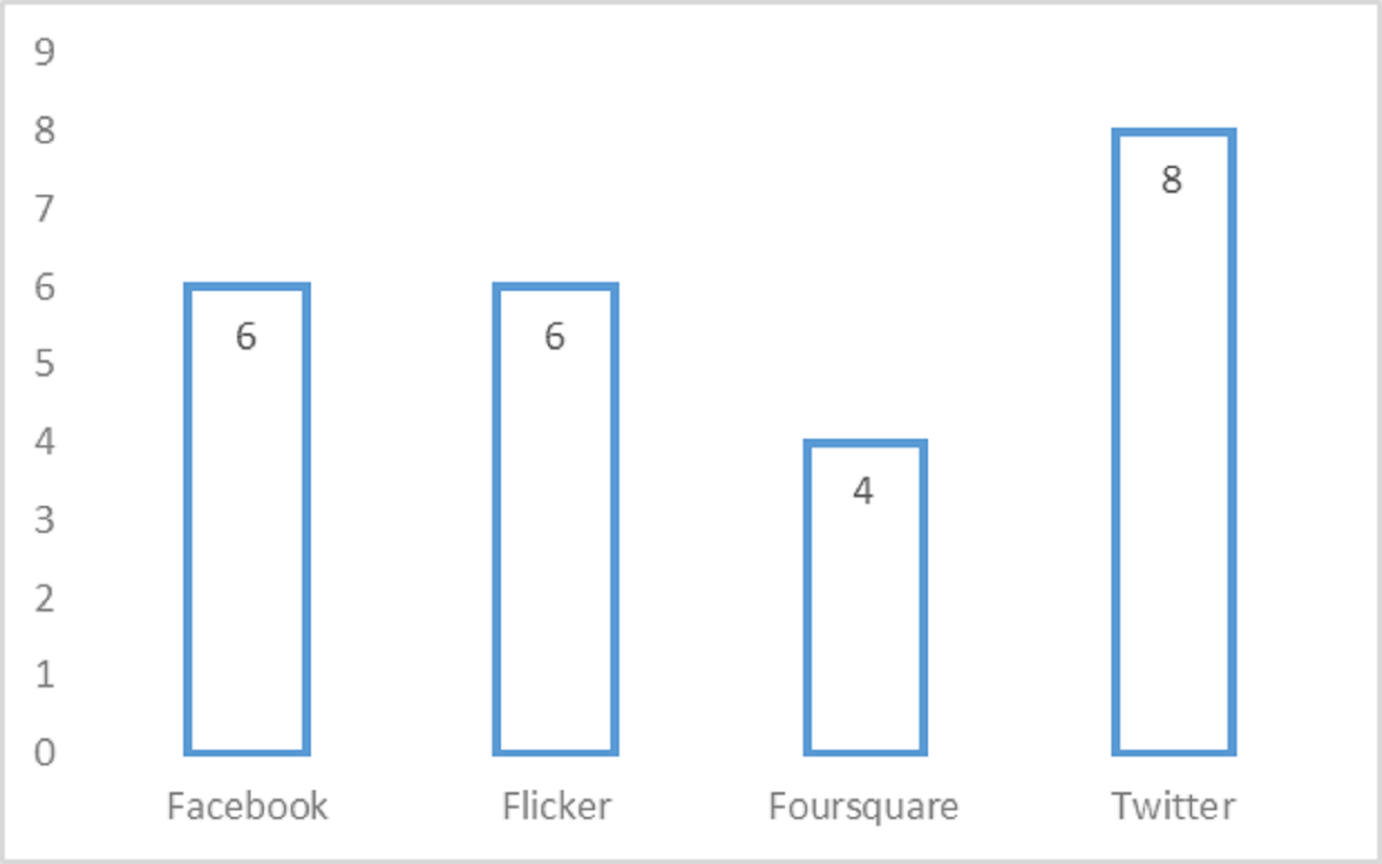
Top four datasets used in domain of DRSN.

**Figure 12:**
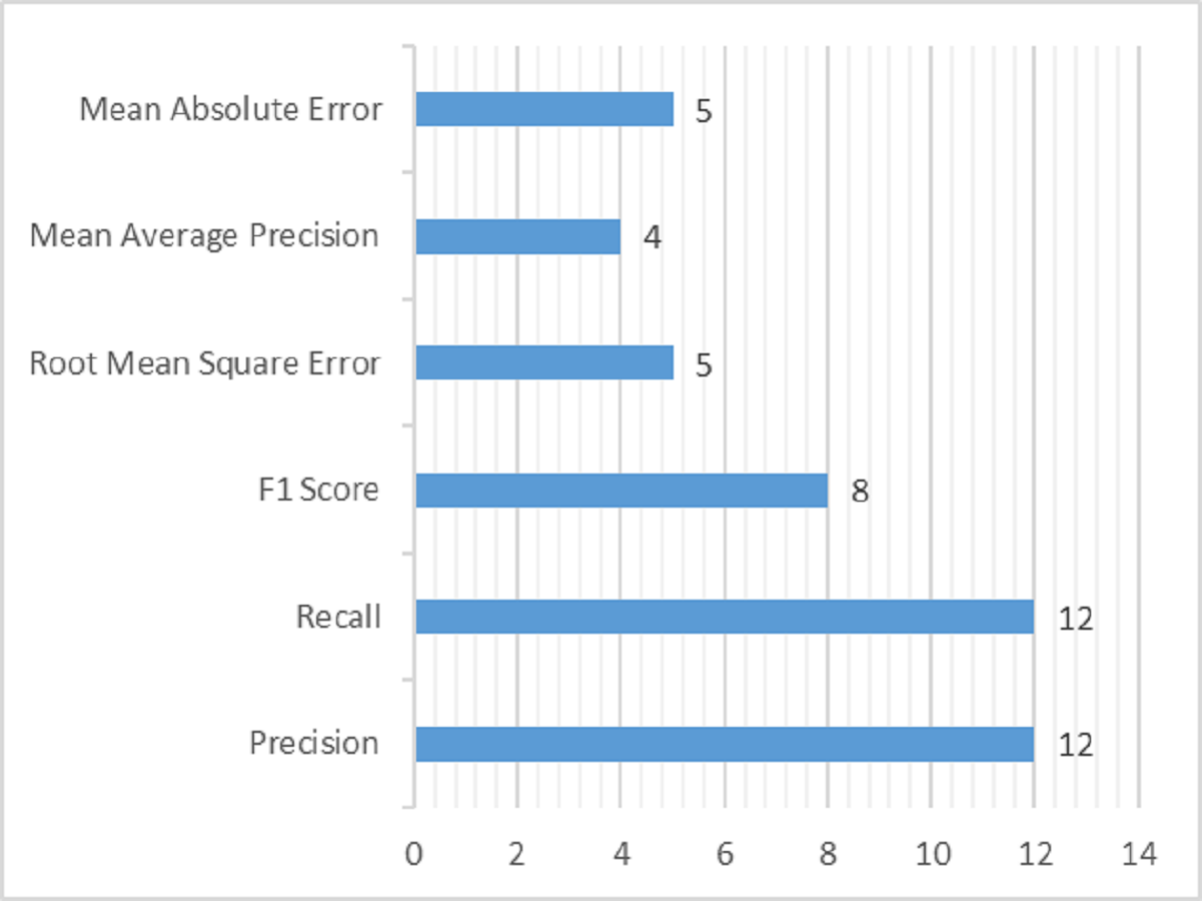
Evaluation metrics used in domain of DRSN.

The datasets and evaluation metrics are an essential part of successful research. The top research trends in the domain of DRSN are shown in Figs. 11 and 12. The complete detail about it is explained deliberately in Table 14.

### RQ 4: What are the limitations of using data mining in RS upon social media websites?

The research process provides novelty and adds contributions to the world. On the other hand, there are certain limitations of each research work. Some researchers explain them explicitly in their work, while others do not. Those who do not mention them, the reader finds it by critically reading the article. The limitations of selected studies are extracted and stated in Table 15. Results showed that even though RS has seen a lot of attention lately, there are still several challenges and possibilities that will influence RS’s future for researchers. Additionally, a variety of strategies that include various aspects can be used to create social recommendation systems that achieve high accuracy. As a result, these limitations lead to research gaps on which future work can be performed.

**Table 15:** Limitations from selected studies in the field of DRSN.

**Ref.**	**Limitations**
Min & Cho (2010)	It does not use any recommendation method like content-based filtering or collaborative filtering. It uses old bayesian network analysis and no proper evaluation has been proposed and needs more evaluation metrics
Zanda, Eibe & Menasalvas (2012)	It requires more evaluation metrics should be included
Majid et al. (2013)	The new user problem and new location problem, so it should consider the content for applying content-based filtering
Fang et al. (2014)	It does not use content-based filtering or collaborative filtering explicitly.
Wang et al. (2014)	More evaluation metrics and proper testing should be applied. It does not use the proper dataset is used for training
Pyo & Kim (2014)	It does not consider social network analysis and is limited to the TV industry
Jiang et al. (2015)	It does not contain is not being used and needs more evaluation metrics
Xu, Chen & Chen (2015)	It only uses the only single method for both mining and recommendation
Wu et al. (2015)	It requires to collect proper data, properly mined and proper recommendation methods need to be implemented
Adeniyi, Wei & Yongquan (2016)	More evaluation metrics can be used along with this content-based filtering can be applied
Jiang et al. (2016)	It uses time-series data only. It can use data other than time series. It does not consider a content-based filtering-based method. Its scope is limited to travel
Zhao, Qian & Xie (2016)	It should use Proper mining and content-based filtering. It should also apply proper evaluation metrics and use content for content-based filtering
Moro, Rita & Vala (2016)	It does not contain is not being used and needs more evaluation metrics
Yu et al. (2016)	It should use Proper mining and CBF. It should also apply proper evaluation metrics
Moro, Rita & Vala (2016)	It requires proper evaluation should be applied. Techniques like content-based filtering or collaborative filtering can be used for recommendation
Wen et al. (2017)	It uses real-time data, which results in higher computation costs. It can also use CF
Shi et al. (2017)	It needs more evaluation metrics and there is need of advance algorithms
Yang & Jiang (2018)	It uses dictionary-based methods for constructing the heterogeneous network based on healthcare and does not consider temporal information, so it does not deal with changing health conditions of patients.
Zhao et al. (2018)	It doesnot consider texual data and do not mine it properly
Sang, Yan & Xu (2018)	It can consider more social media platforms and use their patterns along with data to form social network
Lwowski, Rad & Choo (2018)	The proposed system is not domain independent. It doesnot uses proper evaluation metrics like accuracy , precision, recall etc
Psyllidis, Yang & Bozzon (2018)	It does not uses content based filtering and needs more evaluation metrices
Xu (2018)	It doesnot consider the content of data for recommendations
Alduaiji, Datta & Li (2018)	It requires more evaluation metrics; topic modelling is missing.
Manca, Boratto & Carta (2018)	It requires more metrics and comparison with other techniques
Sun, Fang & Wang (2018)	It should incorporate content-based filtering and it requires more metrics and comparison with other techniques
Yang, Qu & Cudré-Mauroux (2018)	It does not consider data types with continuous values. I require proper mining and content-based filtering. It should also apply proper evaluation metrics
Qin et al. (2018)	It does not consider content for recommendations.
Cui et al. (2018)	It cannot deal with dynamic change and doesnot consider content and social network
Milovanović et al. (2019)	It has no proper evaluation and comparison with other techniques
Zhou et al. (2019)	It can use data other than time series. It doesnot consider It doesnot consider the content based method. Its scope is limited to medical sciences
Zhang et al. (2019)	It does not consider the content they are searching and not making social network
Wu et al. (2019)	It adopted only two basic methods: LINE and Node2vec. It can use collaborative filtering or social network analysis
Ju, Wang & Xu (2019)	It requires evaluation techniques, Comparison with others missing
Iqbal et al. (2019)	It does not consider contextual information. There is no clear description that how different contextual information are combined.
Torres-Ruiz et al. (2020)	It requires evaluation metrics and proper testing should be applied
Dhelim et al. (2020)	It doesnot consider the content of dataset
Nguyen & Cho (2020)	It does not uses CF nd needs more evaluation metrices because results are not properly shown
Ge et al. (2020)	It requires more evaluation metrics can be used along with this CBF can be applied, implementation is missing
Ahmadian et al. (2020)	It requires more advance alogihm like CNN, LDA
Margaris, Vassilakis & Spiliotopoulos (2020)	It needs more evaluation metrics and comparison with other techniques
Shahbaznezhad, Dolan & Rashidirad (2021)	It requires more evaluation techniques and proper comparison with others missing

From the limitations explained in RQ4, it is evaluated that there is a need for a hybrid model. This model will use both contents and statistics to deal with the data. Besides the hybridization of CBF with CF, other methods can be combined with these algorithms. Furthermore, studies have not shown proper methodology to mine or store data that can be further used to provide accurate recommendations. The correct methodology to store data is required for efficient recommendations. Moreover, proper evaluation methods need to be applied and explained deliberately because the studies use the most commonly used evaluation metrics and require proper measurements to validate the work.

## Discussion and Limitation

The previous section discusses the findings and results gathered from research questions. The articles are selected based on particular EDs that are related to DRSN and selected articles are from venues that are JCR listed which covers the Scopus database as well. This section deals with the summarization and discussion of the findings gathered from research questions and formulate a taxonomy that provides an overview of the results.

### Taxonomy order

**Figure 13:**
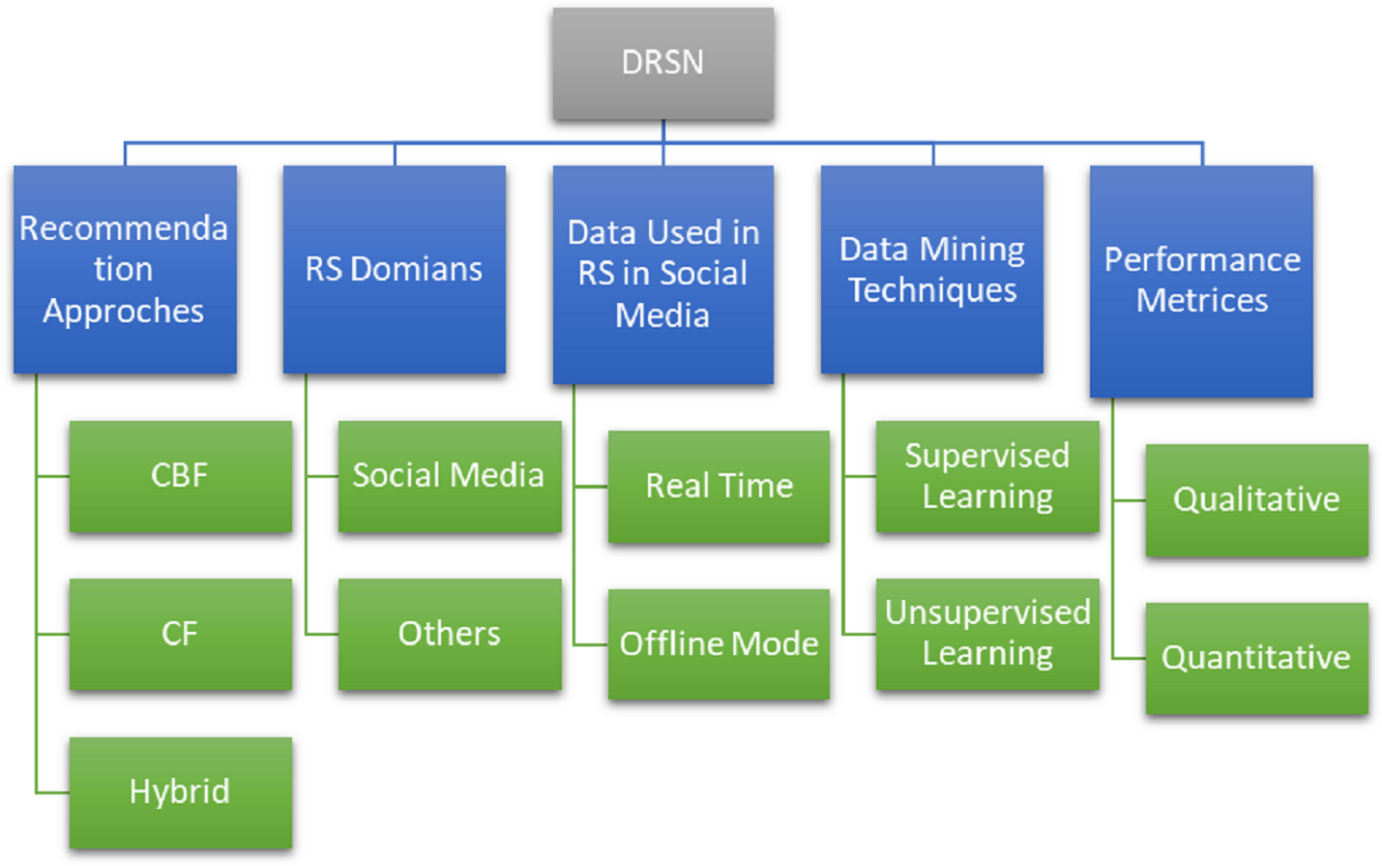
Taxonomy of DRSN perspective.

The main objective of this SLR is to observe the latest trends in DRSN by considering 42 articles. To accomplish this purpose, hierarchical taxonomy of selected articles is formulated and shown in Fig. 13. Several codes are assigned to different methodologies while performing SLR. To to develop a hierarchy. The codes are shown in Table 16. It shows the broader view of SLR. It has inspected advances and challenges in the domain of DRSN in terms of recommendation approaches, recommendation domains, data used in RS in social media, data mining methodologies, and performance metrics. However, its sub-levels are also shown to get a better insight into the DRSN and its types.

**Table 16:** Coding for taxonomy.

**Domain**	**Code**	**Full form**
**Recommendation system schemes**	RS	Recommendation system
	CBF	Content based filtering
	CF	Collaborative filtering
**Data mining and recommendation system methodology on social networks**	DRSN	Data mining based recommendation system on social networks

### Limitation of SLR

The limitation of this SLR includes the unavailability of renowned EDs that are Scopus and Web of Sciences in our region. However, more EDs can be used to extract data.

## General Observation and Future Work

The SLR focuses on four research questions that are discussed in this section along with some future directions.

The RQ1 analyzed that most of the studies are published in IEEE, which implies that IEEE focuses more on recommendation systems and the data mining domain. Furthermore, it was found that China is contributing more to DRNS as the Chinese are progressing to understand current domains better. Furthermore, it has been analyzed that most of the articles were published in 2018 and then in 2020, which shows that social media greatly impacts society. It is very important to utilize this data, thus providing better recommendations to the users. The recommendations provided to the users are based on their search patterns and way of using social media. Different users have different recommendations because each user has it is own modal, patterns, and likings. Therefore, social media plays a significant role in providing recommendations according to the user’s interest.

The RQ2 presents five types of solutions used by studies, namely, framework, method, applications, models, and algorithms. It is analyzed that the most common type of contribution is in the form of methods and then in the form of framework. Furthermore, it was found that most of the studies use the CF algorithm for providing recommendations. CF can be used for data mining and recommendation purposes because it formulates a graph-like structure at the back end. Furthermore, other schemes use CBF and data mining. Some studies use a hybrid algorithmic model by using different methodologies. This hybridization helps in the flexibility of the solution. There are limitations in an algorithm that can be minimized with the help of another process.

The RQ3 presents datasets used by primary studies evaluated in the domain of DRSN and evaluation metrics. Both are an integral part of the research process. Data is the most important thing in social media because all the recommendations are based on that. The dataset can be either online or in offline mode. Most studies use offline mode dataset because it is easy to handle. Moreover, it is evaluated that studies most frequently use the Twitter dataset because Twitter is a big social media platform known for accurate and reliable data. Furthermore, Facebook and flicker are social media platforms dependent on recommendations. Therefore, Twitter, Facebook, and flicker are widely used datasets by different studies. The datasets are extracted from different sources. Different studies use different parameters and diverse sizes of the dataset which make them unique from each other. A high amount of data helps in better results but consumes a lot of processing time. Whereas small data set takes less processing time but it may or may not compromise on quality of results After that, some computations or algorithms are applied to formulate a proposed solution. Therefore, after completing the solution, it is needed to be evaluated. They can be evaluated either qualitatively or quantitatively. Most of the articles focus on quantitative evaluation, in which precision and recall are the most used evaluation metrics.

The RQ4 focuses on the limitations of studies evaluated in the domain of DRSN. It is noted that most studies use hybridization of algorithms to provide recommendations, but they do not hybridize the methodologies or schemes such as CBF with CF. The method hybridizations help to eliminate the limitations of each other. Moreover, more evaluation metrics need to be implemented to validate the results. Furthermore, the limitations in existing studies lead to research gaps. The analysis of studies highlights the gap in existing work that deals with users’ changing interests or new interests along with concept drifts. Furthermore, security-based methodologies are required to deal with social media, ensuring the accuracy of the recommendations. Lastly, the methodology deals with a universal type of recommendations.

Different findings are extracted from this SLR. Four RQs were formulated to examine the methods, approaches, tools, data sets, and performance metrics for recommendation using data mining rules on social media facts and specify an exclusive indication of subject matter. A lot of mixed results can be noted to deal with the issues in DRSN systems. Table 17 shows the RQs that will help formulate a new primary study in DRSN. The details of future directions are given below.

**Table 17:** Research questions for future research directions in the domain of DRSN.

**ID**	**Research question**
**RQ1**	How data mining is dealt with the changing the interest of social data to provide better recommendations?
**RQ2**	What are the criteria’s of managing concepts drift in social media and how mined data will you contribute to getting recommendations accordingly?
**RQ3**	How RS supports security in social media using IoT devices?
**RQ4**	How to ensure the accuracy in recommendations due to a huge number of social media-related data?
**RQ5**	How data mining deals in avoiding universal recommendations in social media as data is in bulk quantity?

### Changing trends in recommend systems

In social media, there are chances of change in user trends depending upon the current situation. This situation will lead to a cold start problem (Lam et al., 2008) and grey sheep problem (Gras, Brun & Boyer, 2016) in which the system will have no prior information about the new trend. The recommended systems require data for providing recommendations. Moreover, the lack of information about new trends shows the limitation, as the RS provides suggestions based on data. The cold start problem can never be eliminated from scratch because it results in data sparsity. The data sparsity makes it difficult during feature extraction and similarity management (Natarajan et al., 2020). In the future, data mining can be used to deal with data based on semantics so that similar data can be incorporated concerning new trends so that the cold-start and grey sheep problem can be minimized.

### Concept drifts in recommend systems

The state-of-the-art recommendation models are not versatile enough to deal with the dynamism problems. Few of the studies overlook the significance of a user’s historical data or experiences during recommendations that affect the results. These results in concept drifts which arise in several ways and at various times, and it should be dealt with accordingly (Klinkenberg & Renz, 1998). For this purpose, certain algorithms should be explored to get the correct research path. Furthermore, the evaluation of concept drift requires real-time due to dynamic user interaction with social media. Therefore, future research requires different assessment methods from state-of-the-art methods, such as precision, recall, and diversity.

### Ensuring security in social media using recommend systems

In social media, security is a major concern to deal with as the data is available online and can be used at any time by any user if proper privacy policies are not applied to the system (Jamiy et al., 2015). Social media is growing at a great pace. Therefore, the system faces certain challenges which are difficult to handle. Criminals that want to breach the security barrier with innovative methods. Traditional methods are unable to identify complex and zero-day attacks. New reliable solutions are required to deal with these issues. Therefore, AI models are used for these purpose and manage the time complexity factor (Shaukat et al., 2020b). Therefore, a real-world and logical solution is required to target social network security assurance. RS should develop a mechanism that verifies the data in the Internet of Things (IoT) devices as data is the main concern of DRSN. The current world is into IoT and moving towards advanced IoT, so the security should not be compromised to maintain the privacy of each individual.

### Accuracy in recommend systems

In RS accuracy of results should be precise to provide correct recommendations. However, there are some loops holes such as some systems that provide high precision on the public data set, but in the case of private data set, the system’s accuracy and privacy of data face clashes. In the case of high private data, there is low accuracy and vice versa, which cannot be improved further (Xu et al., 2020). So, there requires a system that deals with the dynamic interactions of the user. This common recommendation is due to the insufficient response of the user (Xu et al., 2020). Therefore, the system requires optimization and proper interaction of humans with computers to get better results with relevance to accuracy on private data.

### Avoiding universal recommendations

The current era is a time of data explosion as social media produces a large amount of data. The RS was developed to deal with big data. The RS’s have different applications which are evolving over time and thus, resulting in an excess amount of information. However, the system’s performance depends upon the data it is using. Nevertheless, different data sets in recommend systems show different results depending on the domain. Dissimilar data sets are often not compatible with each other, thus resulting in poor recommendations. So, in the future, to increase the usefulness of the system, certain data mining methods using graph theory based on semantics need to be implemented, which eliminates the issue of providing universal recommendations. It formulates graphs so that all the data is linked through semantics and provides better results according to requirements.

## Conclusion

This article performs an SLR in the domain of DRSN. All the abbreviations used in this SLR are shown in Tables A2. A total of 42 articles were investigated and explored. Their publication venues are extracted along with publisher details. A total of 26 articles out of 42 are published in IEEE. Furthermore, chronological distribution as evaluated, stating that most articles were published in 2018 with nine research articles. The proposed solutions in the domain of DRSN are mostly models and frameworks. Moreover, according to the first author of the article, geographical distribution was evaluated, which states that China has the highest ratio of work in this domain. Also, the widely used method by the articles is CF which is used by nine studies. Additionally, most articles contribute to social media, but some work in other domains, such as travel health. The most commonly used data set is Twitter, used by eight studies, and the top trend evaluation metrics in the domain of DRSN are precision and recall, which are used by 12 studies each. Lastly, the limitations show a need for a hybrid model that concatenated both the CBF and CF methods and with or without other methods for providing recommendations. In the future, more research questions can be added to deal with the domain of DRSN to expand the research.

## Appendix

This sections shows the detail of selected articles used in SLR including publication venues, publisher of journals, year of publication, country of first author, type of proposed methodology and their quality score that is calculated on the basis of QAC mentioned in Table 9. The Tables A1 shows the summary.

**Table A1:** Classification of selected articles along with quality assessment score.

References	Publication Venu	Publisher	Year	Country	Proposed solution	QAC1	QAC2	QAC3	QAC4	Total
Min & Cho (2010)	IEEE Transactions On Systems, Man, And Cybernetics	IEEE	2011	Korea	Application	1	0.25	0	0.25	1.5
Zanda, Eibe & Menasalvas (2012)	Expert Systems With Applications	Elsevier	2012	Spain	Method	1	0.75	0.5	0.75	3
Majid et al. (2013)	International Journal Of Geographical Information Science	Taylor Francis	2013	China	Framework	1	1	0.25	1	3.25
Fang et al. (2014)	IEEE Transactions On Multimedia	IEEE	2014	China	Framework	1	0.75	0	0.75	2.5
Wang et al. (2014)	IEEE Transactions On Mobile Computing	IEEE	2014	China	Method + Application	1	0.75	0	1	2.75
Pyo & Kim (2014)	IEEE Transactions On Cybernetics	IEEE	2014	Korea	Framework	1	0.75	0	0.75	2.5
Jiang et al. (2015)	IEEE Transactions On Multimedia	IEEE	2015	China	Framework	1	0.25	0	0.5	1.75
Xu, Chen & Chen (2015)	Neurocomputing	Elsevier	2015	USA	Method	1	0.75	0.75	0.75	3.25
Wu et al. (2015)	IEEE Systems Journal	IEEE	2015	Japan	Method + Application	1	0.25	0	0.5	1.75
Adeniyi, Wei & Yongquan (2016)	Applied Computing And Informatics	Science Direct	2016	China	Method	1	0.75	0.25	1	3
Jiang et al. (2016)	IEEE Transactions On Big Data	IEEE	2016	Boston	Framework	1	0.75	0	0.75	2.5
Zhao, Qian & Xie (2016)	IEEE Transactions On Multimedia	IEEE	2016	China	Framework	1	0.75	0	1	2.75
Moro, Rita & Vala (2016)	Journal Of Business Research	Elsevier	2016	Portugal	Method	1	0.75	0.75	0.75	3.25
Yu et al. (2016)	ACM Transactions On Knowledge Discovery From Data	ACM	2016	China	Framework + Algorithm	1	1	0.25	0.75	3
Moro, Rita & Vala (2016)	Journal Of Business Research	Elsevier	2016	Portugal	Method	1	0.25	0	0.75	2
Wen et al. (2017)	IEEE Transactions On Knowledge And Data Engineering	IEEE	2017	Taiwan	Framework	1	0.5	0.25	0.5	2.25
Shi et al. (2017)	IEEE ACCESS	IEEE	2017	China	Method	1	0.75	0.25	1	3
Yang & Jiang (2018)	IEEE Transactions On Computational Social Systems	IEEE	2018	USA	Method	1	0.5	1	0.5	3
Zhao et al. (2018)	IEEE Transactions On Multimedia	IEEE	2018	China	Framework	1	0.5	0	0.5	2
Sang, Yan & Xu (2018)	IEEE Transactions On Multimedia	IEEE	2018	China	Framework	1	0.5	0	0.5	2
Lwowski, Rad & Choo (2018)	IEEE Transactions On Big Data	IEEE	2018	USA	Method	1	0.25	0	0.25	1.5
Psyllidis, Yang & Bozzon (2018)	IEEE Access	IEEE	2018	Netherlands	Framework	1	0.5	0	0.5	2
Xu (2018)	Information Processing And Management	Elsevier	2018	China	Method	1	0.75	0.75	1	3.5
Alduaiji, Datta & Li (2018)	IEEE Transactions On Computational Social Systems	IEEE	2018	Saudia Arab	Model	1	0.75	0.5	1	3.25
Manca, Boratto & Carta (2018)	Information Systems Frontiers	Elsevier	2018	Canada	Method	1	0.75	0.75	1	3.5
Sun, Fang & Wang (2018)	Personal and Ubiquitous Computing	Springer	2018	China	Method	1	0.75	0	0.5	2.25
Yang, Qu & Cudré-Mauroux (2018)	IEEE Transactions On Knowledge And Data Engineering	IEEE	2018	Switzerland	Framework	1	0.75	0	0.75	2.5
Qin et al. (2018)	IEEE Transactions On Knowledge And Data Engineering	IEEE	2018	Australia	Framework	1	0.75	0	0.5	2.25
Cui et al. (2018)	IEEE Transactions On Systems, Man, And Cybernetics	IEEE	2018	China	Framework	1	0.75	0.5	0.75	3
Milovanović et al. (2019)	Future Generation Computer Systems	Elsevier	2019	Serbia	Method	1	0.5	0.5	0.75	2.75
Zhou et al. (2019)	IEEE Transactions On Computational Social Systems	IEEE	2019	Japan	Model + Framework	1	0.5	0	0.75	2.25
Zhang et al. (2019)	IEEE Access	IEEE	2019	China	Method	1	1	0	0.75	2.75
Wu et al. (2019)	IEEE Access	IEEE	2019	China	Framework	1	0.75	1	0.75	3.5
Ju, Wang & Xu (2019)	Multimedia Tools And Applications	Springer	2019	China	Method	1	0.75	0.75	0.5	3
Iqbal et al. (2019)	IEEE Access	IEEE	2019	Uk	Framework + Algorithm	1	1	0.25	0.75	3
Torres-Ruiz et al. (2020)	Virtual Reality	Springer	2020	Mexico	Method + Application	1	0.25	0.25	0.75	2.25
Dhelim et al. (2020)	IEEE Transactions On Computational Social Systems	IEEE	2020	China	Method	1	0.75	0	0.75	2.5
Nguyen & Cho (2020)	IEEE Access	IEEE	2020	South Korea	Method	1	0.75	0.25	0.75	2.75
Ge et al. (2020)	IEEE Access	IEEE	2020	China	Model	1	0.75	0.5	0.75	3
Ahmadian et al. (2020)	Knowledge-Based Systems	Elsevier	2020	Iran	Method	1	0.75	0.75	1	3.5
Margaris, Vassilakis & Spiliotopoulos (2020)	Information Processing And Management	Elsevier	2020	Greece	Method	1	0.75	0.75	0.75	3.25
Shahbaznezhad, Dolan & Rashidirad (2021)	Journal Of Interactive Marketing	Elsevier	2021	Newzealand	Method	1	1	0.75	0.75	3.5

**Table A2:** List of abbreviations, acronyms and various notations.

**Abbreviation**	**Full form**
ACM	Association of computing machinery
CBF	Content-based filtering
CF	Collaborative Filtering
CNN	Convolutions neural network
DRSN	Data mining-based recommendation systems using social networks
ED	Electronic database
EC	Exclusion criteria
IC	Inclusion criteria
IEEE	Institute of electrical and electronics engineers
JCR	Journal citation report
KKT	Karush–Kuhn–Tucker
KNN	K-nearest neighbour
MAE	Mean absolute error
MAP	Mean average precision
QAC	Quality assessment criteria
RMSE	Root mean square error
RQ	Research question
RS	Recommendation system
SLR	Systematic literature review

The Tables A2 comprises of list of abbreviations, acronyms and various notations.

## Additional Information and Declarations

### Data Deposition

The following information was supplied regarding data availability: There is no raw data for this literature review.
